# Comparison of the clinical efficacy and toxicity of nebulized polymyxin monotherapy and combined intravenous and nebulized polymyxin for the treatment of ventilator-associated pneumonia caused by carbapenem-resistant gram-negative bacteria: a retrospective cohort study

**DOI:** 10.3389/fphar.2023.1209063

**Published:** 2023-08-16

**Authors:** Zhenping Wu, Siying Zhang, Yelin Cao, Qiyu Wang, Keyuan Sun, Xia Zheng

**Affiliations:** ^1^ Department of Critical Care Medicine, The First Affiliated Hospital, Zhejiang University School of Medicine, Hangzhou, China; ^2^ Department of Radiology, The First Affiliated Hospital, Zhejiang University School of Medicine, Hangzhou, China; ^3^ Department of Critical Care Medicine, The People’s Hospital of Jinyun Country, Lishui, China

**Keywords:** ventilator associated pneumonia, polymyxin B, polymyxin E, carbapenem resistant gram-negative bacterial infections, nebulized polymyxin

## Abstract

**Objective:** To investigate the clinical efficacy and toxicity of nebulized polymyxin monotherapy and combined intravenous and nebulized polymyxin for the treatment of VAP caused by CR-GNB. Additionally, among patients treated with nebulized polymyxin monotherapy, we compared the clinical efficacy and toxicity of polymyxin B and polymyxin E.

**Methods:** This study was a single-center, retrospective study. Included patients received aerosolized polymyxin for at least 72 h with or without intravenous polymyxin for the management of CR-GNB VAP. The primary endpoint was clinical cure at the end of polymyxin therapy. Secondary endpoints included AKI incidence, time of bacteria-negative conversion, duration of MV after inclusion, length of stay in ICU, and all-cause ICU mortality.

**Results:** 39 patients treated with nebulized polymyxin monotherapy were assigned to the NL-polymyxin group. 39 patients treated with nebulized polymyxin combined with intravenous use of polymyxin were assigned to the IV-NL-polymyxin group. Among the NL-polymyxin group, 19 patients were treated with polymyxin B and 20 with polymyxin E. The clinical baseline characteristics before admission to the ICU and before nebulization of polymyxin were similar between the two groups. No differences were found between the two study groups in terms of microorganism distribution, VAP cure rate, time of bacteria-negative conversion, duration of MV after inclusion, length of stay in ICU and all-cause ICU mortality. Similarly, survival analysis did not differ between the two groups (χ^2^ = 3.539, *p* = 0.06). AKI incidence was higher in the IV-NL-polymyxin group. When comparing the clinical efficacy and toxicity to polymyxin B and polymyxin E, there was no difference between the two groups in terms of VAP cure rate, time of bacteria-negative conversion, duration of MV after inclusion, length of stay in ICU, SOFA score, CPIS, AKI incidence and all-cause ICU mortality.

**Conclusion:** Our study found that nebulized polymyxin monotherapy was non-inferior to combination therapy with intravenous polymyxin in treating CR-GNB-VAP. Furthermore, we observed no differences in clinical efficacy or related toxic side effects between polymyxin B and polymyxin E during nebulized polymyxin therapy as monotherapy. However, future prospective studies with larger sample sizes are required to confirm these findings.

## Introduction

Ventilator-associated pneumonia (VAP) is one of the most common hospital-acquired infections in intensive care units (ICU), and is defined as pneumonia developing over 48 h after onset of mechanical ventilation (Fernando et al., 2020). In recent years, there has been a worrying increase in infections with drug-resistant Gram-negative bacteria, especially carbapenem-resistant Gram-negative bacteria (CR-GNB). The emergence of carbapenem resistance is a major setback to the ability to effectively treat multidrug-resistant gram-negative bacteria (MDR-GNB) in cases of VAP (David et al., 2019).

Polymyxin is a type of polypeptide antibiotic that has broad-spectrum antibacterial activity against certain bacteria. A report based on the data from the China Antimicrobial Resistance Surveillance System (CARSS) and the China Antimicrobial Surveillance Network (CHINET) indicated that polymyxin maintained high sensitivity to common pathogens of VAP, such as *Escherichia coli*, *Klebsiella pneumoniae*, *Pseudomonas aeruginosa*, and *Acinetobacter baumanii* (Hu et al., 2018). Therefore, polymyxin is recommended as one of the treatment options for patients with CR-GNB infections in VAP.

The polymyxin antibiotics polymyxin E (colistin) and polymyxin B were first approved for clinical use in the late 1950s but were abandoned in the 1970s mainly due to their nephrotoxicity and neurotoxicity. However, given the increasing prevalence of MDR-GNB in hospital-acquired pneumonia (HAP), polymyxin has regained attention as a salvage therapy for gram-negative bacterial infections that cannot be treated by other means. However, intravenous administration of polymyxin is frequently limited by adverse reactions, especially nephrotoxicity, and offers insufficient lung-tissue penetration (Choe et al., 2019). Nebulized polymyxin administration has been suggested as an adjunctive treatment, but the evidence on nebulized polymyxin monotherapy is limited and conflicting. Comparative studies between aerosol polymyxin B and polymyxin E are also needed.

To investigate the clinical efficacy and toxicity between nebulized polymyxin monotherapy and combined intravenous and nebulized polymyxin for the treatment of VAP caused by CR-GNB, we collected clinical data on 78 patients with VAP caused by CR-GNB who accepted nebulized polymyxin treatment with or without intravenous polymyxin. Additionally, among patients treated with nebulized polymyxin monotherapy, we compared the clinical efficacy and adverse reactions of polymyxin B and polymyxin E, in hopes of providing relevant clinical evidence for the treatment of CR-GNB-VAP with nebulized polymyxin.

## Materials and methods

### Study design and patient population

This study was designed as a single-center, retrospective, matched case-control (1:1 ratio) study in the 200-bed intensive care unit (ICU) of The First Affiliated Hospital, Zhejiang University School of Medicine between March 2019 and August 2022. The study protocol was approved by the ethics committee of the institutional review board of The First Affiliated Hospital, Zhejiang University School of Medicine. All critically ill patients older than 18 years, who had received mechanical ventilation for more than 48 h, and who presented with CR-GNB-VAP were eligible for enrollment in the study. Patients’ airway secretions were collected to confirm that CR-GNB was positive and susceptible to polymyxin. Included patients received aerosolized polymyxin for at least 72 h with or without intravenous polymyxin for the management of CR-GNB VAP. Age < 18 years, pregnancy, and septic shock were considered as exclusion criteria. Patients who were treated with intravenous (IV) polymyxin plus nebulized (NL) polymyxin were eligible for the IV-NL polymyxin group. The NL-polymyxin group included those patients who were treated with nebulized polymyxin monotherapy.

### Definition and date collection

Information was extracted from hospital electronic records about patients’ demographic characteristics, primary diagnosis, comorbid conditions, reasons for ICU Admission, days of tracheal intubation before ICU admission, laboratory findings at ICU admission, duration of ICU stay, 28-day survival after ICU admission, and concomitant use of other antibiotics (within 7 days of polymyxin). We also recorded length of intubation, laboratory findings, the Sequential Organ Failure Assessment (SOFA scores), and Clinical Pulmonary Infection Scores (CPISs) of patients within the 24 h before receiving nebulized polymyxin. Data from all patients were reviewed independently by two ICU specialists to check the clinical outcomes in patients of both groups. In the event of a discrepancy, the two reviewers assessed the records again and reached a consensus decision. The response to treatment was assessed at the time of discharge from the ICU or at the end of antimicrobial therapy. The two investigators were not aware of which therapy patients had received.

An episode of VAP was defined as a CPIS higher than six (Zilberberg and Shorr, 2010). Bacteriological samples of tracheal aspirate were taken once a day. A positive tracheal sample was defined as 106 or more colony-forming units (CFU)/ml. Sensitivity to polymyxin was determined by the E test, and the isolated strain was considered sensitive when the minimum inhibitory concentration (MIC) was less than 2 mg/L. Bacteria-negative conversion was defined as sterile culture or absence of the original pathogen in sequential culture after polymyxin treatment (Zha et al., 2020).

At the completion of polymyxin therapy, we classified clinical outcomes on the basis of change in presenting signs and symptoms of infection. The categories were: cure, persistent VAP, recurrence, and superinfection. Cure of VAP was defined as resolution of clinical and biological signs of infection, CPIS less than 6, and negative culture of lower respiratory-tract specimens (if available). Persistent VAP was defined as lack of improvement of clinical and biological signs of infection, CPIS greater than 6, and significant concentrations of CR-GNB persisting in the lower respiratory tract. Recurrence was defined as initial cure of VAP with antimicrobial treatment at day 7 followed by the reappearance of clinical and biological signs of infection, CPIS greater than 6, and significant concentrations of the same pathogen in lower-respiratory-tract specimens. Superinfection was defined as reappearance of VAP caused by pathogens other than the pathogen isolated from lower-respiratory-tract specimens before polymyxin treatment (Lu et al., 2012).

According to the acute-kidney-injury (AKI) guideline from “2012 Kidney Disease: Improving Global Outcomes (KDIGO)”, AKI is defined using the following criteria: an increase in serum creatinine (SCr) of ≥0.3 mg/dL (≥26.5 μmol/L) within 48 h; or an increase in serum creatinine to ≥1.5 times baseline, which is known or presumed to have occurred within the previous 7 days; or a urine volume of < 0.5 mL/kg/h for 6 h (Khwaja, 2012).

### Statistical analysis

Quantitative data were reported as mean ± SD and compared with the Student’s t-test or the Mann–Whitney U test, as appropriate. Qualitative data were expressed as percentages and compared with the Chi-square or Fisher’s exact tests, as appropriate. The risk-association measurement was obtained by stratified analysis and expressed as an odds ratio. Survival analysis was analyzed based on the Kaplan–Meier survival curves and compared with the log-rank test. All tests were two-sided, and a *p*-value < 0.05 was considered to indicate statistical significance. We used IBM SPSS Statistics 20 software for statistical analysis.

### Treatment regimen

Polymyxin E and polymyxin B were available in our hospital. We administered polymyxin E in a daily dose of 300–360 mg colistin base activity (CBA) (∼9–10.9 million IU). The conversion factor was 1 million IU to ∼33 mg CBA. The dose was divided into two and infused over 0.5–1 h at 12-h intervals. We monitored renal function and adjusted the daily dose according to the guideline (Tsuji et al., 2019). The polymyxin B dose of 1.25–1.5 mg/kg (equivalent to 12,500–15,000 IU/kg total body weight) was infused over 1 h every 12 h. Daily maintenance doses of polymyxin B were not adjusted if the patient had renal impairment. For nebulized inhalation of polymyxin, polymyxin B 50 mg was dissolved in 5 mL sterile injection water once every 12 h, and polymyxin E 30 ∼ 60 mg CBA was dissolved in 2∼4 mL normal saline once every 12 h (Tsuji et al., 2019). The medication was nebulized via an ultrasonic vibrating plate nebuliser (Aeroneb Pro^®^ Aerogen Nektar Corporation, Galway, Ireland). This technique required specific settings in order to limit turbulence inspiratory flow: a volume-controlled mode with a tidal volume < 8 mL/kg, respiratory rate at 12 cycles/min, I/E: 1/1, and an inspiratory hold >20%.

## Results

### Enrollment of patients

**FIGURE 1:**
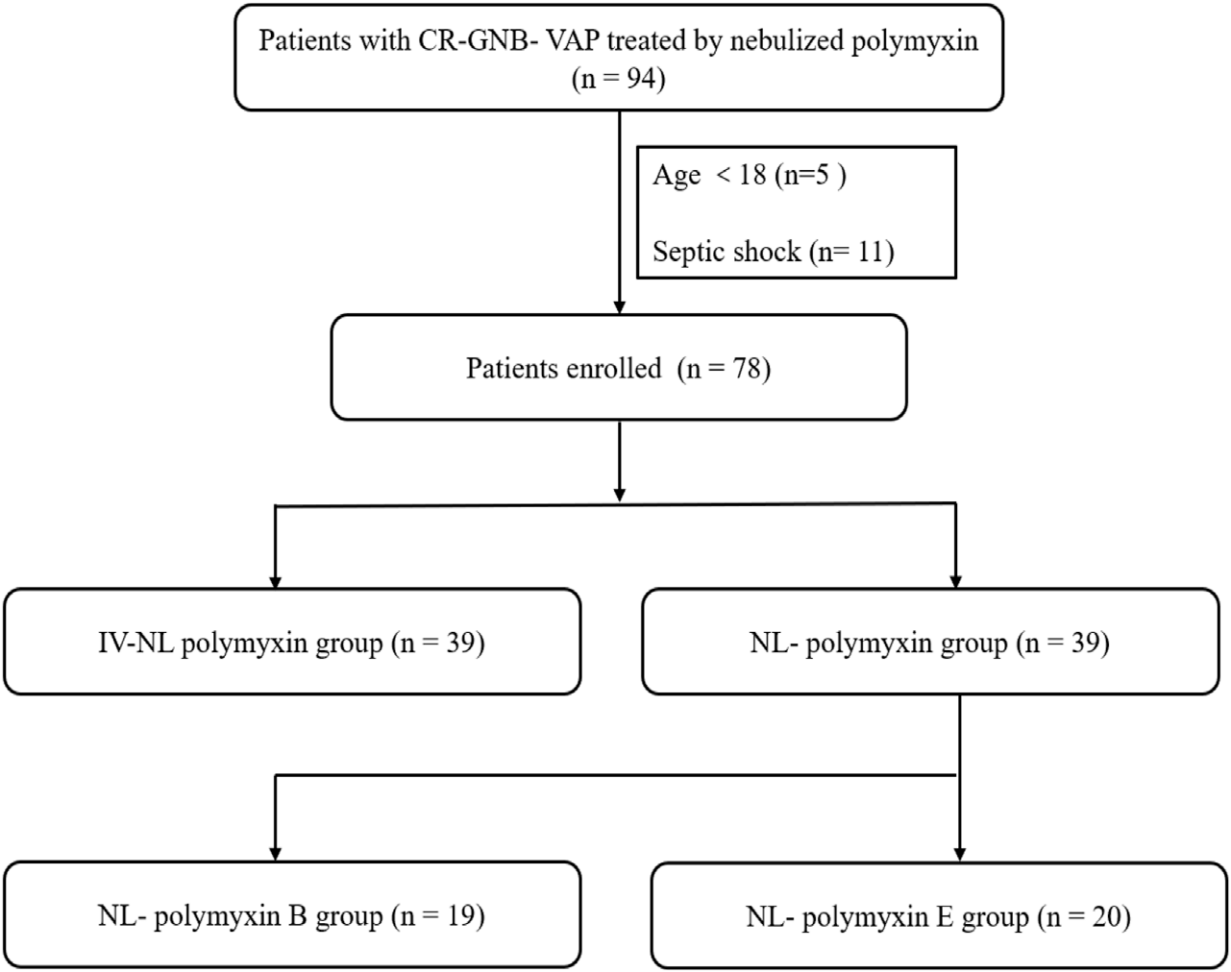
Patients’ flowchart. CR-GNB = carbapenem resistant Gram-negative bacteria, VAP = ventilator-associated pneumonia, IV = intravenous, NL = nebulized.

There were 94 patients with CR-GNB-VAP treated with nebulized polymyxin who were eligible for analysis. 16 people were excluded, including 5 minors and 11 patients with septic shock. 39 patients were assigned to the NL-polymyxin group and treated with nebulized polymyxin monotherapy. 39 patients were assigned to the IV-NL-polymyxin group and treated with both nebulized polymyxin and intravenous polymyxin. In the NL-polymyxin group, 19 patients were treated with polymyxin B and 20 with polymyxin E. The study flow diagram is shown in Figure 1.

### Clinical baseline characteristics before admission to ICU and before nebulization of polymyxin

We analyzed the clinical baseline characteristics of the enrolled patients before admission to ICU. No statistical differences were found between the NL-polymyxin and IV-NL polymyxin groups in terms of gender, age, co-morbidities, or reason for admission to the ICU. We recorded concomitant use of other antibiotics within 7 days of polymyxin; β-lactamases and carbapenems were the most commonly used combination antibiotics during the use of polymyxin, but there was no significant statistical difference between the two groups in concomitant use of other antibiotics (Table 1). After patients were admitted to the ICU, we recorded length of intubation, laboratory findings, SOFA scores, and CPISs of patients within the 24 h before nebulization with polymyxin. As shown in Table 2, intubation time and laboratory findings were the same between the two groups. Similarly, we found that there was no statistical difference between the two groups in terms of SOFA scores or CPISs.

**TABLE 1:** Clinical baseline characteristics before admission to the ICU.

Variables	Total	NL-polymyxin	IV-NL polymyxin	P-value
n	78	39	39	
Male (%)	42 (53.84%)	23 (58.97%)	19 (48.72%)	0.364
Age (years)	61.2 ± 15.9	59.9 ± 18.0	62.5 ± 13.6	0.123
Co-morbidities (n, %)				
Solid malignancy	7 (8.97%)	5 (12.82%)	2 (5.13%)	0.428
Diabetes	21 (26.92%)	7 (17.95%)	14 (35.90%)	0.074
Hypertension	25 (32.05%)	12 (30.77%)	13 (33.33%)	0.808
Cirrhosis	4 (5.13%)	1 (2.56%)	3 (7.69%)	0.608
Chronic kidney diseases	2 (2.56%)	2 (5.13%)	0 (0%)	0.474
Coronary disease	17 (21.79%)	9 (23.77%)	8 (20.51%)	0.784
Underlying lung diseases	26 (33.33%)	11 (28.21%)	15 (38.46%)	0.337
Reasons for admission (n, %)				
Internal disease	47 (60.26%)	23 (58.97%)	24 (61.54%)	0.817
Multiple trauma	8 (10.26%)	4 (10.26%)	4 (10.26%)	1.0
Surgical diseases	23 (29.49%)	12 (30.77%)	11 (28.21%)	0.804
Concomitant use of antibiotics (n, %)				
Quinolones	13 (16.67%)	7 (17.95%)	6 (15.38%)	0.761
β-lactamases	64 (82.51%)	32 (82.51%)	32 (82.05%)	1.0
Carbapenem	40 (51.28%)	19 (48.72%)	21 (53.85%)	0.651
Aminoglycosides	6 (7.69%)	1 (2.56%)	5 (12.82%)	0.089
Vancomycin	24 (30.77%)	13 (33.33%)	11 (28.21%)	0.624

**TABLE 2:** Clinical baseline characteristics in the ICU before nebulization with polymyxin.

Variables	Total	NL-polymyxin	IV-NL polymyxin	P-value
Intubation days	12 (6.20)	11 (6.20)	12 (6.21)	0.711
WBC(×109/L)	11.95 ± 7.09	11.06 ± 7.23	12.84 ± 6.92	0.270
PLT (×109/L)	199.67 ± 134.31	198.41 ± 119.96	200.92 ± 148.97	0.935
CRP (mg/L)	80.82 ± 73.97	65.49 ± 64.04	96.15 ± 80.64	0.067
PCT (ng/mL)	2.67 ± 8.13	1.69 ± 2.82	3.65 ± 11.14	0.291
ALT (U/L)	45.62 ± 54.03	54.92 ± 61.56	36.31 ± 44.14	0.129
TbiL (μmol/L)	27.96 ± 67.09	34.20 ± 91.61	21.72 ± 25.48	0.415
INR	1.37 ± 1.31	1.27 ± 0.49	1.47 ± 1.79	0.504
Cr (μmol/L)	89.73 ± 74.73	86.74 ± 84.98	92.72 ± 63.84	0.727
SOFA score	7 (4.10)	6.5 (3.9)	7 (4.10)	0.372
CPIS	7 (6.8)	7 (6.7.25)	7 (6.8)	0.058

### Microorganism distribution

**FIGURE 2:**
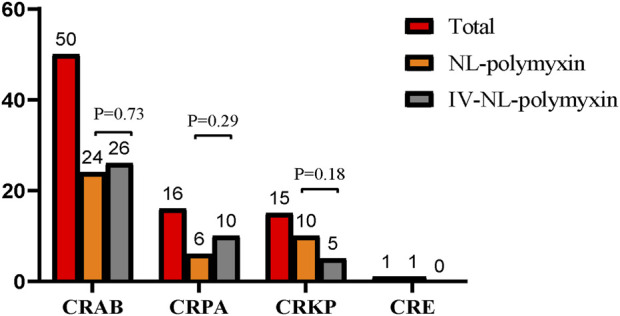
Microorganism distribution in the study groups. Among the cultured drug-resistant bacteria, CRAB accounted for the highest proportion, followed by CRPA and CRKP, and CRE was rarely found in respiratory specimen culture. No significant statistical difference was found in microorganism distribution between the study groups. CRAB = Carbapenem-resistant *Acinetobacter baumannii*, CRPA = Carbapenem-resistant *Pseudomonas aeruginosa*, CRKP = Carbapenem-resistant *Klebsiella pneumoniae*, CRE = Carbapenem-resistant Enterobacterales.

From all 78 cases, a total of 82 strains of CR-GNB were cultured from sputum specimens when CR-GNB-VAP was diagnosed. Three patients had more than one CR-GNB cultured from the same respiratory sample, two of which had CRAB and CRKP, while the other had CRAB and CRPA. Among the cultured drug-resistant bacteria, CRAB accounted for the highest proportion, followed by CRPA and CRKP, and CRE was rarely found in respiratory specimen culture. No significant statistical difference was found in microorganism distribution between the study groups (Figure2).

### Assessment of therapeutic efficacy and toxicity

**FIGURE 3:**
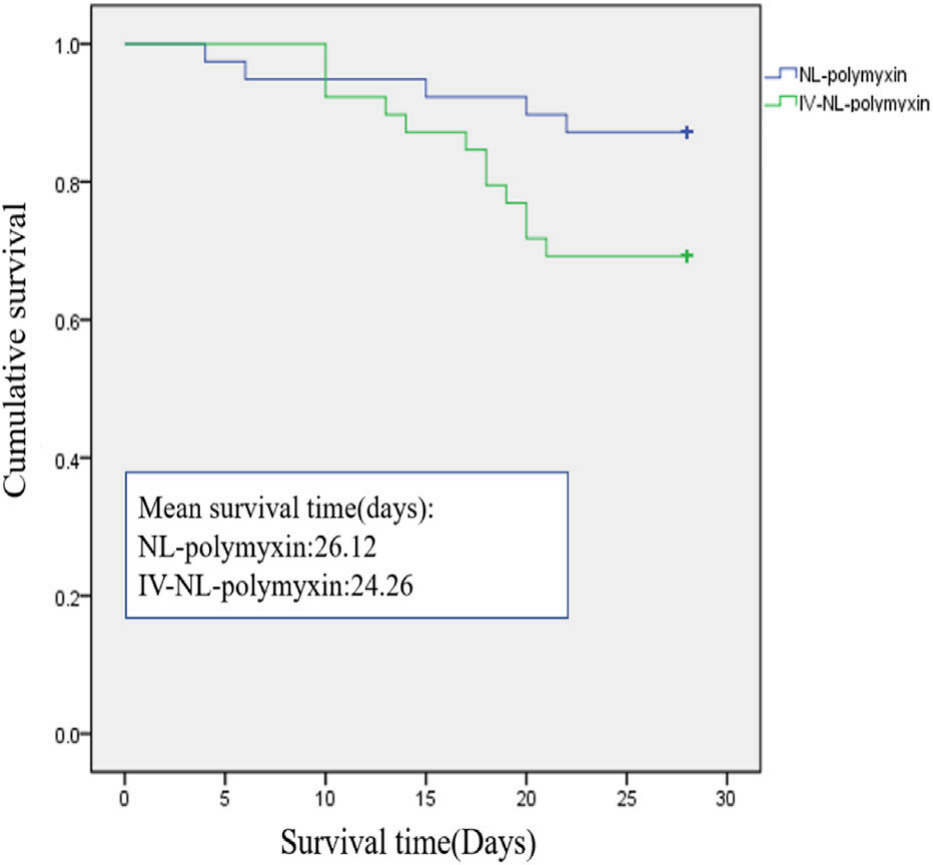
Ventilator-associated pneumonia–related mortality in the two treatment groups. There was no difference between NL-polymyxin and IV-NL-polymyxin groups in terms of survival analysis (χ^2^ = 3.539, *p* = 0.06).

All patients were evaluated for clinical efficacy and related toxicity of polymyxin on the 7th day after the start of polymyxin treatment. 29 of the 39 patients in the NL-polymyxin group had achieved clinical cure at the time of evaluation, and 28 of the 39 patients in the IV-NL-polymyxin group had achieved clinical cure. There was no significant difference in the clinical cure rate of VAP between the two groups. There were eight patients with persistent VAP in the NL-polymyxin group, including three cases caused by CRAB, two cases caused by CRPA, two cases caused by CRKP, and one case caused by CRE. In the IV-NL-polymyxin group, there were seven patients with persistent VAP, including four cases caused by CRAB, two cases caused by CRPA, and one case caused by CRKP. The rates of persistent VAP in the two groups were similar. Patients with recurrence of VAP and superinfection of VAP were less numerous. In the NL-polymyxin group, there was one patient with recurrence of VAP, and the pathogen was CRE. In the IV-NL-polymyxin group, there was also one patient with recurrence of VAP, and the pathogen was CRAB. With regard to superinfection of VAP, there were three cases in the IV-NL-polymyxin group and one in the NL-polymyxin group; the pathogen cultured from all these patients at the first diagnosis of VAP was CRAB. There was no difference between the two groups in terms of time of bacteria-negative conversion, duration of MV after inclusion, length of stay in ICU, AKI incidence, or all-cause ICU mortality. Similarly, survival analysis was the same between the NL-polymyxin and IV-NL-polymyxin groups (χ^2^ = 3.539, *p* = 0.06) (Figure 3). We found no difference between the two groups in terms of SOFA scores or CPISs on the 7th day after starting polymyxin therapy (Table 3).

**TABLE 3:** Assessment of therapeutic efficacy and toxicity.

	NL-polymyxin	IV-NL polymyxin	P-value
Cure of VAP (n, %)	29 (74.36%)	28 (71.79%)	0.799
CRAB	16	20	
CRPA	6	6	
CRKP	9	3	
Persisting of VAP (n, %)	8 (20.51%)	7 (17.95%)	0.774
CRAB	3	4	
CRPA	2	1	
CRKP	2	2	
CRE	1	0	
Recurrence of VAP (n, %)	1 (2.56%)	1 (2.56%)	
CRAB	0	1	
CRE	1	0	
Superinfection of VAP (n, %)	1 (2.56%)	3 (7.69%)	
CRAB	1	3	
Time of bacteria negative conversion	5 (3.5.5)	3 (3.5)	0.202
Duration of MV after inclusion	9 (7.21)	10 (6.20)	0.662
Length of stay in ICU	14 (7.26)	11 (7.20)	0.940
CPIS	4 (3.7)	5 (4.7)	0.403
SOFA	6 (3.9)	7 (4.13)	0.198
AKI (n, %)	4 (10.71%)	10 (25.93%)	0.042
All-cause ICU mortality (n, %)	11 (28.21%)	17 (43.59%)	0.157

Among all patients, 23 patients had pre-existing renal injury before using polymyxin; 11 of these were in the NL-polymyxin group and 12 in the IV-NL-polymyxin group. There were also two cases of chronic kidney disease in the NL-polymyxin group, and we excluded all of these before comparing post-treatment AKI incidence. In the NL-polymyxin group, there was a total of 28 cases, with three cases of AKI (accounting for 10.71%), including one patient receiving vancomycin during polymyxin treatment and one receiving amikacin. In the IV-NL-polymyxin group, there were a total of 27 cases, nine of which developed AKI (accounting for 33.33%), including 1 case receiving amikacin in addition to polymyxin. There was a statistical difference in AKI incidence between the two groups, with a higher incidence in the IV-NL-polymyxin group (Table 3). It was difficult to accurately track adverse bronchospasms reactions during nebulization with polymyxin due to our retrospective data-analysis approach, so we did not analyze this in depth. However, no patients were required to discontinue treatment due to adverse airway reactions caused by nebulization.

### Comparison of efficacy and AKI incidence between nebulized polymyxin B and nebulized polymyxin E

We divided the NL-polymyxin group into a nebulized polymyxin B group and nebulized polymyxin E group. There were 19 cases in the nebulized polymyxin B group, with 15 cases cured and one case developing AKI. The nebulized polymyxin E group had 20 cases, with 14 cases cured and two cases developing AKI. There was no difference between the two groups in terms of VAP cure rate, time of bacteria-negative conversion, duration of MV after inclusion, length of stay in ICU, SOFA score, CPIS, AKI incidence, or all-cause ICU mortality (Table 4).

**TABLE 4:** Efficacy and AKI incidence in the nebulized polymyxin B and nebulized polymyxin E groups.

	Polymyxin B	Polymyxin E	P-value
Cure of VAP	15 (78.95%)	14 (70.00%)	0.716
Time of bacteria negative conversion (day)	5 (3.5)	3.5 (3.6)	1
Duration of MV after inclusion (day)	7 (7.24)	9.5 (6.25.20.5)	0.843
Length of stay in ICU (day)	14 (7.24)	15 (7.28)	0.593
CPIS	4 (4.6)	4.5 (2.25.7)	0.921
SOFA	6 (2.75.8.25)	7 (2.10)	0.831
AKI (n, %)	1 (5.26%)	2 (10%)	0.520
All-cause ICU mortality (n, %)	4/19 (21.05%)	7/20 (35%)	0.480

## Discussion

In our study, we primarily compared the efficacy and safety of nebulized polymyxin alone *versus* nebulized polymyxin in combination with intravenous administration in treatment of CR-GNB-VAP. The clinical baseline characteristics before admission to the ICU and before nebulization with polymyxin were similar between the two groups. There was no statistical difference between the two groups with regard to SOFA scores or CPISs, reflecting to some extent the fact that the overall severity of the disease and the severity of pneumonia in the two groups were similar. Based on our results, we found no difference in the distribution of microorganisms between the NL-polymyxin and IV-NL polymyxin groups, and CRAB was the most common pathogen in both groups, which is consistent with the distribution of VAP multidrug-resistant pathogens in the ICU (Fernando et al., 2020).

VAP caused by MDR bacteria, especially CR-GNB, is a challenging clinical problem that involves high mortality rates and significant healthcare costs. While polymyxin has been widely recommended for treatment of these infections, its systemic use is associated with toxic side effects, and lung-tissue penetration is often suboptimal. Therefore, there has been increasing interest in the use of nebulized colistin to treat VAP caused by MDR bacteria. Early experiences with nebulized polymyxin were in patients with cystic fibrosis and bronchial superinfection (Littlewood et al., 1985). Over the next decade, polymyxin was administered intratracheally to prevent or treat lung superinfection and VAP in critically ill patients (Rouby et al., 1994; Markou et al., 2003; Karvouniaris et al., 2015), In terms of using nebulized polymyxin to prevent VAP, there is no conclusive evidence that it is successful, and there is a risk of introducing antibiotic-resistant organisms when antibiotics are used widely in critically ill patients in the ICU. Thus, nebulized polymyxins are not currently recommended for prevention or treatment of VAP. Some guidelines mention the use of nebulized polymyxins to treat VAP caused by MDR bacterial infections. The 2016 Infectious Disease Society of America (IDSA) and American Thoracic Society (ATS) guidelines gave a weak recommendation for using aerosolized antibiotics only in situations in which the infectious pathogen is susceptible to polymyxin or aminoglycosides (Kalil et al., 2016). In contrast, the European Respiratory Society does not recommend the use of inhaled antibiotics at all; instead, their recommendation is to avoid the use of nebulized antibiotics to treat VAP (Rello et al., 2017; Torres et al., 2017). The conflicting recommendations stem from the weak evidence in published studies regarding the efficacy of aerosolized antibiotics.

There is a paucity of data on the efficacy of nebulized polymyxin as a monotherapy for pneumonia caused by MDR-GNB (Kang et al., 2014; Abdellatif et al., 2016; Kim et al., 2017); however the majority of studies have found that nebulized polymyxin as a combination therapy can improve VAP treatment efficacy compared to intravenous monotherapy. Fewer studies compare the efficacy of nebulized polymyxin alone and combined nebulized and intravenous polymyxin. Our results suggest that treatment with nebulized polymyxin monotherapy can achieve similar clinical VAP cure rates to combined nebulized and intravenous polymyxin. A meta-analysis performed on 12 studies published between 2005 and 2016 reported the effectiveness of nebulized CMS as a monotherapy for treating respiratory tract infections caused by MDR-GNB and/or GNB that are only susceptible to colistin (CMS). The clinical and microbiological success rate was 70% (Vardakas et al., 2018), an efficacy similar to that in our study. We found that the time of bacteria-negative conversion, duration of MV after inclusion, length of stay in ICU, and all-cause ICU mortality were also similar in the two groups. This suggests the feasibility of using nebulized polymyxin alone for the treatment of VAP. The advantage is that reducing the use of intravenous polymyxin can reduce drug toxicity and treatment costs. However, in the clinical diagnosis and treatment process, clinicians often adopt a plan based on intravenous use of polymyxin, either alone or combined with nebulized polymyxin. Our results may be related to the exclusion criteria adopted for our enrolled patients, as we excluded patients with septic shock. Larger, randomized clinical trials comparing the efficiency of nebulized polymyxin alone and nebulized combined with intravenous polymyxin in treating CR-GNB-VAP may be more convincing, but the ethics of such trials may be challenging.

Neuromuscular toxicity, nephrotoxicity, and bronchoconstriction are the most common adverse events associated with polymyxin administration. Polymyxin-induced neuropathy and myopathy are rarely seen. Nephrotoxicity is the most common side effect observed with both colistin and PMB, and most frequently results from intravenous administration of CMS (Zavascki and Nation, 2017). In this study, we compared the toxic side effects in two groups of patients. As expected, the incidence of AKI in the combined-therapy group was significantly higher than that in the nebulization-monotherapy group, due to the drug exposure in the combined group being significantly higher; this increased the burden on the kidneys. Immunostaining studies performed in rodents have shown predominant accumulation of polymyxins in proximal tubular cells of the renal cortex (Velkov et al., 2010). The resultant high intratubular colistin concentrations cause mitochondrial damage, loss of cytoplasmic membrane potential, apoptosis, and cell cycle arrest (Eadon et al., 2013). However, it is worth noting that nebulized polymyxin monotherapy can still result in renal injury; the incidence of AKI in the NL-polymyxin group was 10.71% in our study, while other studies on inhaled colistin monotherapy for respiratory-tract infections in adults without cystic fibrosis found an incidence of 20% (Vardakas et al., 2018). AKI may be related to the concomitant use of other drugs that cause renal injury. In this study, patients in the nebulization group who developed AKI were also using other potentially nephrotoxic drugs, including one case with combined use of vancomycin and polymyxin and one case with combined use of amikacin. Adverse bronchospasm reactions during nebulization with polymyxin were difficult to accurately count due to the retrospective data analysis. However, as we mentioned above, no patients were required to discontinue treatment due to adverse airway reactions caused by nebulization. Because the subjects of this study were VAP patients in the ICU, most of whom were under sedation and analgesia, no further statistical analysis was performed on neuromuscular toxicity.

Both polymyxin B and polymyxin E were available in our hospital, and both are recommended for treating MDR-GNB VAP according to our Chinese national expert consensus on the use of polymyxin (Chinese and Chinese, 2019). We distinguished between the polymyxin B and polymyxin E groups of patients treated with nebulized polymyxin, and found no significant differences in clinical efficacy or related toxic side effects. Due to differences in access to polymyxin B in various countries and regions, there are relatively few international studies on nebulized polymyxin B. Some studies have investigated nebulized polymyxin B as an adjunctive treatment with intravenous polymyxin B in VAP patients, and found that combination treatment can achieve better clinical efficacy (Hasan et al., 2021; Zhou et al., 2021; Lin et al., 2022). We have searched the relevant literature, and there appears to be no clinical trial comparing the results of polymyxin B and polymyxin E nebulized therapy for VAP. Theoretically, the currently used polymyxin E is CMS as a precursor drug, which has no antibacterial activity. It can take effect only after being converted into polymyxin E *in vivo*. This process takes a long time, and the drug-conversion rate at the target site is relatively low. However, polymyxin B, when administered in its active form, can quickly achieve a therapeutic effect in the lungs, has a strong bactericidal effect, and can neutralize endotoxins. The therapeutic effect of polymyxin B may be better than that of polymyxin E. However, we found no relevant differences in this study, possibly because of the high local drug concentration caused by nebulization therapy, which far exceeds the bactericidal concentration. PK/PD studies of polymyxin nebulization in in vitro and *in vivo* experiments have confirmed this idea (Lin et al., 2017; Zheng et al., 2020). We also cannot rule out the possibility that the similarity is due to the small sample size or to data bias caused by retrospective analysis.

Indeed, one limitation of this study is its retrospective design, which may have led to selection bias and confounding factors. In addition, the sample size was relatively small, which may limit the generalizability of the findings. Due to limited data, we did not subdivide CR-GNB into a single strain, but rather compared it as a whole. It is possible that different bacteria respond differently to nebulized polymyxin. Finally, we were unable to obtain accurate information on the concentration of polymyxin in the lining fluid of alveolar epithelial cells after nebulization. If PK/PD related data could be obtained, our results would be more convincing.

## Conclusion

In conclusion, our study found that nebulized polymyxin therapy as a monotherapy was non-inferior to combination therapy with intravenous polymyxin in treating CR-GNB-VAP. We observed no differences in clinical efficacy or related toxic side effects between polymyxin B and polymyxin E resulting from nebulized polymyxin monotherapy. However, future prospective studies with larger sample sizes would be required to confirm these findings.

## Data Availability

The raw data supporting the conclusion of this article will be made available by the authors, without undue reservation.
